# Priority-setting in TB programme planning: guiding an equitable and effective response

**DOI:** 10.5588/ijtldopen.26.0258

**Published:** 2026-08-11

**Authors:** D. Pedrazzoli, F. Mavhunga, T.A.b.m. Islam

**Affiliations:** World Health Organization, Geneva, Switzerland.

**Keywords:** tuberculosis, resource allocation, strategic planning, health financing, health governance

## Abstract

Despite being preventable and curable, TB remains a major infectious killer. Effective control requires prevention, early diagnosis, proper treatment, and the ability to address socio-economic risk factors. With limited resources, countries must prioritise interventions amid evolving epidemiology, technologies, and health system demands. To support this, the WHO’s policy brief, ‘Priority-setting in tuberculosis programme planning’, outlines structured and participatory decision-making approaches. It frames priority-setting as an ongoing governance function, not a one-time response to scarcity, helping align resources with population needs, promote equity, and strengthen integration of TB services within primary health care and universal health coverage.

Despite being preventable and curable, TB remains a leading infectious killer, with more than 10 million people falling ill and over a million dying annually.^1^ Chronic underfunding of health systems remains a challenge in many low- and middle-income countries (LMICs), where government health expenditure is limited relative to competing national priorities.^2^ TB programmes continue to face financing gaps, with resources falling short of national strategic plan needs.^1^ In 2024, total funding for TB services in LMICs reached only US$ 5.7 billion – just 26% of the US$ 22 billion annual global target.^1^ Recent shocks (including COVID-19 disruptions, economic downturns, and reduced international funding) threaten to reverse progress towards ending TB.^3,4^ Modelling suggests funding reductions could result in up to 2 million additional deaths and 10 million cases between 2025 and 2035.^5,6^

Priority-setting is an inherent component of health sector planning, which helps to determine which interventions to deliver, for whom, and in what order, when resources are constrained.^7^ The recent funding pressures have renewed attention on the need for explicit prioritisation in TB programme planning.^8^ In many settings, priority-setting has been informal and reactive, shaped by historical allocations, donor requirements, or immediate operational pressures rather than deliberate, inclusive processes.^9^ The WHO policy brief ‘Priority-setting in tuberculosis programme planning’^10^ frames priority-setting as a systematic, evidence-informed and people-centred practice. It promotes a shift from implicit decision-making to structured, criteria-based processes that are transparent, inclusive, and evidence-informed. This shift is particularly timely as countries prepare for the Global Fund allocation cycle and gradually transition away from donor funding towards domestic financing, requiring a clearer alignment of resources with national priorities. Priority-setting must also be dynamic. The epidemiology of TB continues to evolve, including changes in incidence, risk groups, and drug resistance patterns. At the same time, innovations, for example, in diagnosis and treatment, offer new opportunities but require deliberate choices about scale-up. Health systems are also undergoing broader transformation, with increasing emphasis on primary health care (PHC)-oriented systems and universal health coverage (UHC), requiring TB services to be better integrated into service delivery platforms.^11–13^ Institutionalising priority-setting can help ensure TB responses remain agile and aligned with broader health system reforms.

## THE APPROACH TO PRIORITY-SETTING FOR TB PROGRAMMES

Effective priority-setting requires balancing epidemiological and programmatic evidence, cost-effectiveness, social equity, and feasibility within existing system capacities. It requires country-led, context-specific decision-making grounded in evidence and aligned with national health strategies.^7^ Equity and human rights should be deliberately considered in making choices, ensuring access for underserved populations is not sacrificed in efforts to focus only on interventions that are easier to implement or that yield more immediate gains. Maintaining quality of care and avoiding fragmentation are essential for sustainable impact.^10^ Health system capacity, including infrastructure, workforce, supply chains, financing, and information systems, ultimately determines what interventions can be implemented effectively and sustained over time.^7^

Frameworks exist to support structured priority-setting for health services. A practical approach is the 3Ds – ‘data, dialogue, and decision’, which emphasises evidence (data); stakeholder engagement (dialogue); and criteria-based selection of priorities (decision).^7,14^ When integrated into national strategic planning,^15,16^ these approaches can strengthen governance and stakeholder trust through transparency, participation, and accountability.^15^ Effective priority-setting also involves navigating difficult trade-offs. Decision-makers must weigh short-term gains against long-term system strengthening, ensure efficiency does not undermine equity, and maintain quality of care within system constraints. Meaningful stakeholder engagement, particularly of affected communities, is essential to the legitimacy of these choices, even if it can be operationally demanding.^17^ Making such trade-offs explicit strengthens feasibility and credibility.

## TRANSLATING PRIORITIES INTO ACTION: EXAMPLES FROM TB PROGRAMMES

The WHO policy brief outlines key considerations for translating priority-setting into programmatic action, emphasising that decisions should be comprehensive, context-specific, and grounded in health system realities. Rather than prescribing a fixed set of interventions, it offers a framework to guide how countries identify, sequence, and justify priorities across the TB response. A practical way to operationalise these principles is through the 3Ds approach:

- Data: Countries use epidemiological, programmatic, and economic evidence to identify gaps and opportunities across the TB cascade of care.^18^ For example, large detection gaps may prompt investment in digital case-based surveillance.
- Dialogue: Inclusive engagement with stakeholders, including government sectors, implementers, and affected communities, ensures priorities reflect societal values, feasibility, and equity. For example, in settings where access barriers disproportionately affect rural or marginalised populations, stakeholder dialogue may highlight the need to prioritise decentralised and community-based TB services.
- Decision: Transparent, criteria-based processes guide selection and sequencing of interventions, balancing impact, equity, and feasibility. For example, in settings with a high burden of TB infection among close contacts, programmes may prioritise scaling up TB preventive treatment.

Importantly, priority-setting should take a comprehensive view across all pillars of the End TB strategy,^19,20^ while remaining adaptable to country context and aligned with broader health system capacity. In this way, prioritisation translates strategy into action.

### Health sector reforms

TB programmes do not operate in isolation; their success depends on alignment with broader national priorities for PHC, UHC, and health financing reform. Priority-setting should be embedded in benefit package design, financing, and service delivery reforms. When integrated in this way, priority-setting helps ensure that essential TB services are guaranteed to all, that affected people are protected from financial hardship, and that TB interventions are delivered within PHC-oriented health system. In the context of growing fiscal pressure on TB programmes, structured priority-setting becomes particularly crucial in helping countries navigate funding shocks without undermining impact or equity. While many of the key financing decisions lie beyond the remit of TB programmes alone, national TB programmes play a critical role in informing prioritisation within broader health sector processes. Within this context, three complementary approaches can help sustain progress^21^:

1. Inform resource mobilisation efforts: TB programmes can support broader government efforts to expand fiscal space by clearly articulating priority interventions and their expected impact, including through budget prioritisation and engagement with financing partners.^22^
2. Enhance efficiency and impact: TB programmes can improve both technical efficiency (doing things right), for example, through pooled procurement and streamlined supply chains, and allocative efficiency (doing the right things),^23^ by investing in high-impact interventions such as targeted TB screening and shorter regimens for TB prevention and treatment.
3. Safeguard critical services: TB programmes can maintain life-saving interventions during fiscal shocks by applying transparent, criteria-based triage approaches, including for ongoing treatment, essential diagnostics, and drug supply continuity.

Beyond periods of constraint, priority-setting is a proactive governance tool that strengthens decision-making, transparency, and accountability. Applying explicit, evidence-informed criteria to define ‘must-do’, ‘should-do’, and ‘could-do’ interventions helps maintain essential services under pressure, guide phased expansion as resources evolve, and support engagement with financing and planning processes. As countries are increasingly required to justify priorities across multiple platforms, including national strategic plans, health sector reforms, and external funding processes, institutionalised priority-setting strengthens both resilience and strategic ambition, regardless of the level or stability of available financing.

## CONCLUSION

The WHO policy brief on priority-setting in TB programme planning offers national TB programmes a practical roadmap for strengthening decision-making, improving accountability, and ensuring that TB responses are equitable, impactful, and sustainable. It comes at a critical moment for global TB efforts. As eligible countries prepare for the next Global Fund cycle, strengthening prioritisation is essential. Priority-setting is not simply a technical exercise; it is a core governance function that shapes how countries translate commitments into action using limited resources. When implemented through inclusive, transparent, and evidence-informed processes, priority-setting enhances the coherence and effectiveness of TB programmes, supports alignment with PHC and UHC reforms, and ensures that resources are used where they have greatest impact. In practice, priority-setting inevitably involves difficult choices and, at times, unavoidable sacrifices. Not all needs can be met simultaneously; decisions often entail weighing which consequences are more acceptable in a given context, whether in terms of populations reached, services prioritised, or pace of scale-up. Making these trade-offs explicit, and engaging stakeholders, including affected communities, in the process, is therefore critical. When communities understand the rationale for decisions and have a voice in shaping them, they are more likely to accept necessary compromises and support their implementation. Such engagement can also help mobilise local resources and initiatives, as seen in community-driven approaches to supporting TB patients in some settings.^24^

Ending TB requires more than good intentions and new tools. It requires clear, deliberate choices under strong governance. By embedding structured priority-setting into routine planning and policymaking, countries can strengthen their TB responses today while building the resilient health systems needed for tomorrow.
